# Discoloration Potential of Biodentine: A Systematic Review

**DOI:** 10.3390/ma14226861

**Published:** 2021-11-14

**Authors:** Monika Slaboseviciute, Neringa Vasiliauskaite, Saulius Drukteinis, Luc Martens, Sivaprakash Rajasekharan

**Affiliations:** 1 Institute of Dentistry, Faculty of Medicine, Vilnius University, Zalgirio 115, 08217 Vilnius, Lithuania; monika.slaboseviciute@gmail.com (M.S.); n.vasiliauskaite27@gmail.com (N.V.); 2 Department of Paediatric Dentistry, Ghent University School of Oral Health Sciences, B-9000 Ghent, Belgium; luc.martens@ugent.be (L.M.); sivaprakash.rajasekharan@ugent.be (S.R.)

**Keywords:** Biodentine, discoloration, staining potential

## Abstract

The aim of this systematic review is to investigate the teeth discoloration potential of Biodentine. An electronic search in six databases (PubMed, Cochrane Library, LILACS, SCIELO, Web of Science, and Scopus) was conducted by three independent reviewers to identify eligible articles. The following search terms were used: ((discolo*, staining potential, color, colour, or spectrophotomet*), (teeth or tooth), and (Biodentine)). Methodology following the PRISMA (Preferred Reporting Items for Systematic Reviews and Meta-Analysis) guidelines was adopted for this investigation. At the end of the selection process, 30 articles were identified as eligible, of which 14 in vitro studies were included in this systematic review. Nine of the included studies evaluated the discoloration potential of Biodentine in the presence of blood. Within the limitations of this review, teeth discoloration using Biodentine is highly expected when material is placed in direct contact with blood during dental procedures. In the absence of blood, Biodentine causes less teeth color changes than MTA-based materials, but it is still unclear what clinically relevant results could be expected regarding the discoloration frequency and intensity induced by Biodentine.

## 1. Introduction

Nowadays, success in endodontic treatment is presented by functioning teeth without clinical symptoms and radiographic evidence of periapical inflammation [1]. However, in the anterior region, another essential aspect of the treatment is acceptable aesthetics. Discoloration of tooth structure, secondary to the use of endodontic cements, is currently the most common concern regarding endodontic treatment [2,3,4,5]. Materials, such as mineral trioxide aggregate (MTA) and calcium silicate-based cements (CSC), are widely used for various restorative and endodontic treatment procedures, such as indirect and direct pulp capping, pulpotomy, apexification, regenerative endodontics, sealing of perforations, and retrograde root fillings [4,6,7,8].

The first commercial CSC introduced to the market, grey MTA, was composed of calcium, silica, and bismuth oxide [6]. Many studies reported that grey MTA leads to tooth discoloration [2,9,10,11,12,13,14,15]. To overcome this limitation, white MTA (wMTA) has been developed with a reduction of aluminum, magnesium, and ferrous oxides. Reduction of ferrous oxide resulted in the elimination of the aluminoferrite phase, which is responsible for the gray color of grey MTA [9]. However, it has been shown that particularly bismuth oxide can cause tooth discoloration and possible mechanisms of it are related to the oxidation of the iron content [2], oxidation of bismuth oxide [16,17], and interference of bismuth oxide with dentin collagen [9].

Biodentine^TM^ (Septodont, Saint Maur des Fosses, France), further referred as Biodentine, is a CSC which was introduced as a “dentine replacement” or “dentine repair” material and is claimed to overcome some of the limitations of MTA [18]. The powder of Biodentine contains calcium carbonate, tricalcium silicate, and zirconium oxide as radiopacifier, while the liquid is water-based calcium chloride as a setting accelerator [6,16]. The physical properties of Biodentine, compared with the MTA, were improved by modifying its powder composition and adding accelerator, softener, and shortened setting time as well as the replacement of bismuth oxide as a radiopacifier with zirconium oxide [6,11,19]. Biodentine has high biocompatibility and bioactivity, with enhanced properties, such as a quick setting time, homogeneity, perfect sealing ability, and high compressive strength [6,20]. In addition, it has been claimed that Biodentine causes less coronal discoloration than MTA [13,21,22,23,24,25,26,27,28], as zirconium oxide did not result in discoloration, and, importantly, did not influence the hydration of the material [29,30]. Therefore, the lack of bismuth oxide in Biodentine significantly reduces the discoloration potential of the material [10,31]. Unlike MTA, Biodentine has the advantage of being more similar to natural tooth color and preventing the discoloration induced by the translucency of material through hard tissues of the teeth [5]. In a systematic review of 23 in vitro studies evaluating discoloration potential of the various CSCs, it was concluded that Biodentine has one of the lowest staining potentials among materials evaluated that was not noticeable to the human eye (ΔE < 3.3) [10], whereas ΔE is a value of measuring the color change of the subject between different time points, used in all studies mentioned and included in this systematic review.

However, only a few studies evaluated Biodentine-induced discoloration in vivo. The Parinyaprom et al. [32] study evaluated Biodentine-induced discoloration after direct pulp capping and none of the teeth in the Biodentine group had visually perceptible grey discoloration in photographs. The Abuelniel et al. [33] study is in agreement with these findings—no discoloration was observed after pulpotomy in traumatized anterior immature teeth up to 18 months, although it is not clear how discoloration was evaluated [33]. In the study of Linu et al. [34], visually no discoloration was observed in the Biodentine group, but it should be mentioned that diffuse calcification of the pulp chamber was observed in 23.1% of the cases treated with Biodentine. On the other hand, Aly et al. [35], Haikal et al. [36], and Uesrichai et al. [37] reported contradictory results. After a revascularization procedure using a double antibiotic mixture (metronidazole and ciprofloxacin) for root canal disinfection, after a one-year observation period, visual discoloration was identified in 1 out of 13 teeth (7.69%) in the Biodentine group [35]. After pulpotomy using Biodentine, slight visual discoloration was observed in 17% of teeth [36] and in another study perceptible grey discoloration was observed in 27% (32) of teeth treated with Biodentine during the mean follow up period of 32.3 months [37]. 

Therefore, due to the current contradictory information about the discoloration potential of Biodentine, the aim of this systematic review is to summarize the existing scientific data on the discoloration potential of Biodentine used for human teeth crown restoration and endodontic procedures. The scientific articles were selected to answer the following question: what is the expected human teeth hard tissue staining potential of Biodentine?

## 2. Materials and Methods

The following review was performed in accordance with the indications of PRISMA (Preferred Reporting Items for Systematic Reviews and Meta-Analysis) criteria [38]. The PICO (population, interventions, comparisons, and outcomes) was defined as follows: Do human permanent teeth subjected to endodontic or crown restoration procedures (population) using Biodentine (intervention) compared to teeth that did not have contact with Biodentine due to endodontic or crown restoration procedures (comparison) show changes in color of hard tissue expressed as mean color change values at various time periods (outcome)?

### 2.1. Search Strategy

Studies were identified through electronic search of selected databases. No publishing year limit was applied on the literature search and the last search was run on 21 July 2021.

Six databases were searched by two reviewers (M.S. and N.V.): PubMed, Cochrane Library, LILACS (added ibecs, bbo-dentistry databases), SCIELO, Web of Science, and Scopus with the following search terms (staining potential, spectrophotomet*) and MeSH terms (discolo*, color, colour, teeth, tooth, Biodentine): ((discolo* OR staining potential, color, colour, or spectrophotomet*), (teeth or tooth) and (Biodentine)). The search included articles written only in the English language.

After selecting articles for inclusion in this review, additional manual search of reference lists of these studies was conducted to identify possible relevant publications.

### 2.2. Inclusion and Exclusion Criteria

The abstracts of in vivo and in vitro studies were screened by M.S. and N.V., who determined whether the publications measured discoloration potential with its relationship with the application of Biodentine. The eligibility criteria for full text review and inclusion were applied at the start of screening and no article was excluded without assessment. The study population of potentially eligible articles should be human samples only (hard tissue of permanent human teeth) and only reliable color measuring devices (spectrophotometer and spectrocolorimeter,) or color determination methods should be used. Also, only studies that conducted color determination prior to treatment or had control groups regarding Biodentine were included. 

The publications that did not use permanent human teeth tissue or used primary human teeth and had only visual color determination methods, or assessment methodology that was not precisely reported, were excluded from this review. 

### 2.3. Study Selection and Data Extraction

(1) The titles and abstracts of identified publications were manually screened for potential duplicate studies and relevance of the current systematic review. Duplicates, along with articles unrelated to the topic, were excluded. 

(2) Using inclusion criteria, full-text assessment of studies was performed. Only articles that satisfied the selection parameters were included in the analysis. 

Steps 1 and 2 were performed independently by two reviewers (M.S. and N.V.). In case of disagreement, the third author (S.D.) was consulted until consensus was reached. Cohen’s κ-value for inter-rater agreement between first and second reviewers was calculated. Significant characteristics (first author’s name, year of publication), study design, sample size, type of teeth, materials investigated, methodology, irrigation protocol, method used for evaluation of teeth discoloration, and results were extracted from each of the included studies. Data extraction and the methods of reporting were performed according to the Cochrane Handbook for Systematic Reviews of Interventions, Chapter 7 [39].

In case of missing or unclear data, the corresponding authors were contacted by email two times over 1 month to obtain relevant information.

### 2.4. Risk of Bias Assessment 

Risk of bias of the included studies were evaluated by two independent authors (M.S. and N.V) in three domains using a modified Cochrane Collaboration Risk of Bias (RoB) tool. Lack of agreement between the reviewers was solved by a third examiner (S.D.).

Studies with at least one domain of high risk of bias were classified as “high risk of bias”. Studies with no domains of high risk of bias but with at least one domain of unclear risk of bias were classified as “unclear risk of bias”, while studies with all domains evaluated as low risk of bias were classified as “low risk of bias”. 

## 3. Results

**Figure 1:**
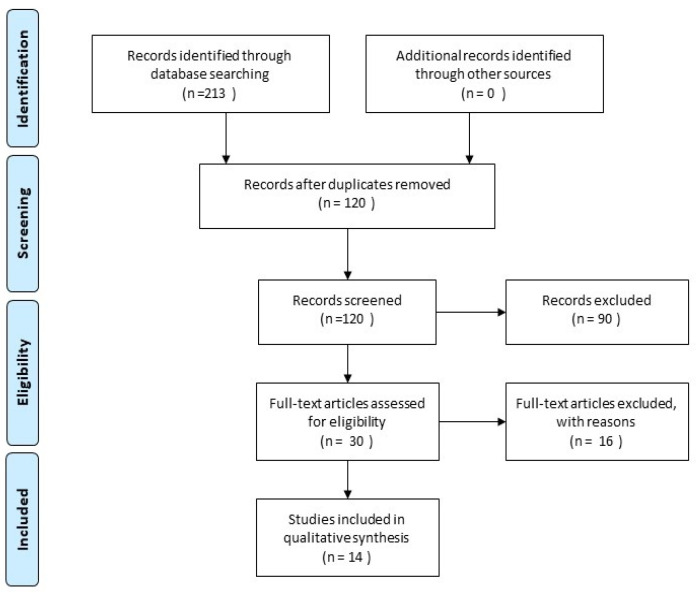
A search flowchart according to the Preferred Reporting Items for Systematic Reviews and Meta-Analyses statement.

Cohen’s κ-value for inter-rater agreement was 0.87. The electronic search identified a total of 213 studies, then, 93 duplicate studies were removed, resulting in 120 studies for screening. After analyzing the titles and abstracts, 30 publications were selected for full-text reading. After complete reading of the articles, fourteen in vitro studies were included in this systematic review after contacting authors for missing information: three authors confirmed that permanent teeth were used in their publications and two provided missing data regarding results, whereas authors of six articles did not provide necessary information, therefore, those publications were excluded. The selection strategy is shown in the PRISMA flow chart (Preferred Reporting Items for Systematic Reviews and Meta-Analyses) [38] (Figure 1).

### 3.1. Risk of Bias Assessment

**Figure 2:**
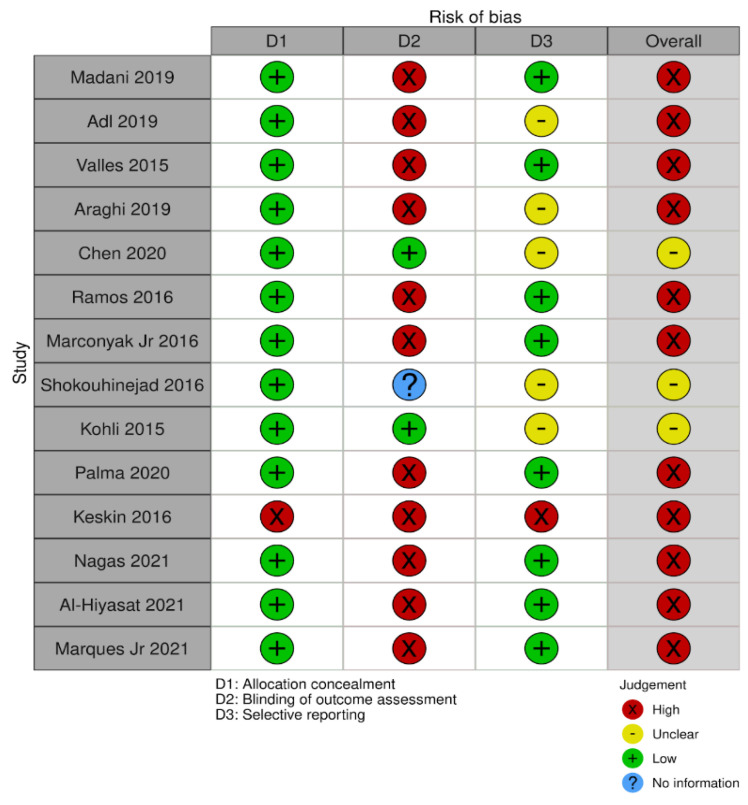
Risk of bias summary according to the Cochrane Handbook for Systematic Reviews of Interventions.

Eleven studies presented high risk of bias and three studies were classified as having unclear risk of bias (Figure 2). The main shortcomings were related to blinding. Only two studies performed blinding procedures of the operator. All studies, except one, presented low risk of bias in allocation concealment domain. Regarding selective reporting, only one article was evaluated as having high risk of bias. Despite this, the overall risk of bias could still be considered as high.

### 3.2. Study Characteristics 

The publications included for the analysis were those of Valles et al. (2015) [27], Kohli et al. (2015) [24], Shokouhinejad et al. (2016) [23], Ramos et al. (2016) [22], Marconyak Jr. et al. (2016) [28], Keskin et al. (2016) [40], Madani et al. (2019) [21], Araghi et al. (2019) [41], Adl et al. (2019) [42], Palma et al. (2020) [26], Chen et al. (2020) [25], Nagas et al. (2021) [14], Al-Hiyasat et al. (2021) [13], and Marques Jr. et al. (2021) [12]. 

The data extracted from included studies are shown in tables: Table 1. Data Extracted from the Included Studies, Table 2. The Changes in Color (∆E) Measured for Materials Tested at Different Time Intervals.

Overall, a total of 243 teeth in the Biodentine group were evaluated. The most common type of teeth tested were anterior (110), followed by premolars (73), a combination of anterior and premolars (30) and mandibular third molars (30). 

The most common method for color change evaluation was spectrophotometry, used in six studies [12,13,21,27,41,42], followed by spectrophotometry used with a digital camera [14,24,25,28,40], colorimetry [22], spectroradiometry [23], and only a digital camera [26].

Follow-up time varied between studies ranging from 1 month (Keskin et al. [40]) to 2 years (Nagas et al. [14]).

Color changes were measured by calculating ∆E values in all studies. Eleven publications [12,13,14,21,22,24,25,26,28,40,42] indicated human eye perceptible color change ranging from ∆E1.2 to ∆E3.7, whereas three articles [23,27,41] did not provide numeric information about ∆E thresholds, which were taken as perceptible to the human eye.

From 14 included studies, nine [12,13,14,22,25,26,27,41,42] provided ∆E values, while five articles [21,23,24,28,40] did not present numeric ∆E values.

### 3.3. Discoloration Potential in the Presence of Blood

Six articles (Madani et al. [21], Adl et al. [42], Chen et al. [25], Shokouhinejad et al. [23], Palma et al. [26]), Al-Hiyasat et al. [13]) evaluated the discoloration potential of Biodentine in the presence as well as absence of blood; two articles (Marques Jr. et al. [12] and Keskin et al. [40]) compared the material blood contamination with the control group without blood and one article (Ramos et al. [22]) compared Biodentine without blood with the positive control group with blood.

Madani et al. [21], Chen et al. [25], and Palma et al. [26] found that all materials in their studies, including Biodentine, showed significantly visible and more severe teeth discoloration in the presence of blood compared to the groups with saline after 1 week (*p* < 0.05). This is in agreement with the study of Shokouhinejad et al. [23], where Biodentine contamination with blood significantly increased ∆E values (*p* < 0.05), although perceptible color change threshold was not specified. Al-Hiyasat et al. [13] presented the same results, as the Biodentine/blood group exceeded perceptibility threshold at all time intervals compared to the Biodentine/saline group. Moreover, they drew a conclusion that Biodentine was the material most affected by the presence of blood in case of color changes (*p* < 0.001) when compared to other materials tested. What is more, the increase of ∆E at 6 months was almost double for Biodentine when tested with blood.

On the contrary, in the study of Adl et al. [42], Biodentine contamination with blood was not associated with greater teeth discoloration compared to the Biodentine/saline group. They found a perceptible color change in the Biodentine/blood group at 3 months (∆E 4.20 ± 2.89); however, it was not statistically significant for the Biodentine/saline group.

Marques Jr. et al. [12] evaluated Biodentine discoloration potential only in the presence of blood, as the revascularization procedure was simulated and found clinically perceptible; statistically significant (*p* < 0.001) color changes starting one month after material placement and lasting at all time periods were tested compared to the control group with temporary zinc-oxide based cement. This is partly in agreement with the study of Keskin et al. [40], where Biodentine contaminated with blood exhibited perceptible color changes after 24 h already and was observed at all time points compared to the baseline, which was recorded before the placement of the material.

Ramos et al. [22] evaluated Biodentine discoloration potential in the absence of blood; however, control groups consisted of the negative group with dry cotton pallets and the positive group with cotton pallets moistened with blood. Control groups with blood demonstrated the highest and statistically significant (*p* < 0.05) ∆E changes in Ramos et al. and in all studies included where contamination with blood was investigated (Adl et al. [42], Chen et al. [25], Palma et al. [26]), Al-Hiyasat et al. [13]).

### 3.4. Discoloration Potential in the Absence of Blood

From articles where quantitative values of ∆E were not provided, studies of Marconyak Jr. et al. [28] and Kohli et al. [24] were partly in agreement with each other. Kohli et al. [24] found no color change exceeding the perceptibility threshold induced by Biodentine without blood at any time interval, whereas Marconyak Jr. et al. [28] found no color change in the Biodentine/saline group compared to the negative control from 7 to 60 days; however, noticeable discoloration was seen at day 1 in their study. On the contrary, Madani et al. [21] stated that all materials without blood, including Biodentine, demonstrated visible color changes (ΔE ≥ 3.3) at all time intervals, which increased over time. This is in agreement with results of Shokouhinejad et al. [23], where color changes in all experimental groups without blood, including Biodentine/saline, increased over time, therefore, the correlation of time and ∆E was significant (*p* < 0.05).

Nine remaining articles, which presented quantitative values of ∆E, are listed in Table 2. Studies of Adl et al. [42], Chen et al. [25], Valles et al. [27], Araghi et al. [41], and Nagas et al. [14] are in agreement regarding the discoloration potential of Biodentine without blood in all time intervals evaluated. Adl et al. [42] and Chen et al. [25] found that the Biodentine/saline group did not reach ∆E thresholds of 3.7 and 3.3, respectively, at any time interval. Meanwhile, according to Valles et al. [27], there were no significant differences between the Biodentine/saline and control groups at any different time measuring points (*p* > 0.05). Similar results were gathered from the study of Araghi et al. [41] and Nagas et al. [14], as color change induced by Biodentine/saline was not significant at anytime interval. Although Nagas et al. [14] observed discoloration in the Biodentine/saline group at three months, it was followed by a smaller and insignificant (*p* > 0.05) increase, which did not reach the perceptibility threshold (∆E ≥ 3.3) compared to the negative control group. Also, Al-Hiyasat et al. [13] found that the Biodentine/saline group did not reach a clinically perceptible color change level (ΔE ≥ 3.7) at any time of the evaluation period (24 h, 1 week, 1, 3, and 6 months).

On the contrary, Ramos et al. [22] and Palma et al. [26] found perceptible-to-human-eye teeth color changes in Biodentine without blood experimental groups at 6, 52 weeks and 1 week, 6 months, respectively. It should be noted that in the Ramos et al. [22] study, the Biodentine-induced perceptible (∆E ≥ 2.3) teeth discoloration did not reach discoloration values in comparison to the negative control group.

## 4. Discussion

Dentist’s perception of tooth color may be easily influenced by surrounding illumination, angles between the illuminant, the object, and the eye; clothing, even make-up, and the chromatic perception of the individual [43,44]. Therefore, electronic color determination devices, such as a spectrophotometer, colorimeter, spectroradiometer and digital camera were investigated and compared to visual methods since the early 1970s [43,45,46,47,48,49]. Nevertheless, not all researchers confirm the clinical superiority of electronic color determination methods [45,48,49], its accuracy and, most importantly, repeatability over the visual method are proven [46,47,50]. For these reasons, it was decided to exclude studies where only a more subjective visual color determination method was used from this systematic review.

All included studies used L*a*b* color space defined by the International Commission on Illumination (CIE) to calculate the differences in color changes, on which ISO (the International Organization for Standardization) guidance on color measurement in dentistry is based [51]. L* represents the darkness—lightness coordinate (from 0 [black] to 100 [white]), a* the chromaticity between green (−) and red (+), and b∗ the chromaticity between yellow (+) and blue (−). ΔE describes the color difference between the initial time point (after material placement) and each subsequent time point measurement. The formula used for ΔE calculation [52] and used in all articles was:

ΔE = [(L1 − L0)2 + (a1 − a0)2 + (b1 − b0)2]1/2

However, there was high heterogeneity regarding the interpretation of ΔE in the included studies. One of the articles [12] regarded changes of color perceptible to human eye when ∆E ≥ 1.2, one [22] when ∆E ≥ 2.3, six articles [14,21,25,26,28,40] when ∆E ≥ 3.3, and three [13,24,42] when ∆E ≥ 3.7. Meanwhile, three studies [23,27,41] did not provide numeric ∆E values and thresholds, from which color changes were regarded as statistically significant at all. These differences may have a negative impact on the interpretation of data, as it makes comparison between studies and its results more difficult and inaccurate.

Among 14 articles, included in this review, six studies [12,13,21,27,41,42] used spectrophotometers for evaluation of color changes, followed by five studies where spectrophotometer was used with digital camera [14,24,25,28,40], one used a colorimeter [22], one, a spectroradiometer [23] and another one, only a digital camera [26]. Therefore, it can be suggested that the spectrophotometer is the most common and accessible device in dental research for colorimetric evaluation. However, although it is considered to be a reliable tool for the determination of color changes, it has some drawbacks as well, such as their application being impaired by the convex surface of the teeth and possibly complicating the correct placing of the probe, which is essential for precise results [44]. Another disadvantage is its difficult application in clinical settings, caused by complex and expensive equipment; therefore, there is a lack of high-quality in vivo studies [49].

Studies using bovine teeth were excluded from this systematic review in order to eliminate possible bias. Comparing chemical composition of enamel and dentin with other mammal species, Teruel et al. [53] concluded that human and bovine teeth demonstrate greatest similarity among the species analyzed. Based on the study of Camargo et al. [54], dentine exposed at the incisal surface of human and bovine teeth presented similar clinical and micro-morphological aspects, represented by surfaces with equivalent numbers of open dentinal tubules. The Schilke et al. [55] study demonstrated that the numbers of tubules in human and bovine coronal dentine were not significantly different, as there were no significant differences regarding the number of tubules per square-millimeter and their diameters in corresponding coronal dentine layers of human deciduous and permanent molars, as well as in the dentine of bovine incisors. These results suggest that the dentine of bovine incisors’ can be a suitable substitute for human molar dentine for adhesion studies, given the similarity of the collagen organic matrix, as both are composed of collagen type I [56]. However, the dentine of bovine incisor roots appears less suitable due to its significantly higher tubule density. Also, some studies have shown differences between human and bovine teeth structure. The findings of Yassen et al. [57] and Ortiz-Ruiz et al. [58] claim that inconsistent data exist regarding whether bovine teeth can be considered an appropriate substitute for human teeth. Differences of morphological and chemical composition, as well as different physical properties between human and bovine teeth were found and must be considered [57,58]. As tooth discoloration is a result of tubular penetration of material components, it may influence the degree of discoloration observed in bovine versus human teeth [55]. Due to the similar reasons, studies with primary human teeth were excluded from this systematic review, as the dentine of primary teeth has greater tubule density and diameter, and a decreased amount of peritubular dentin and presence of microcanals, which may have influence on discoloration frequency, intensity, and clinical relevance [59].

It is well known that blood can cause discoloration of teeth hard tissue and exacerbate MTA-like materials and calcium silicate-based materials staining potential [2,3,22,23,25,26,27,42,60,61]. Marin et al. [60] concluded that the main cause of discoloration in non-infected traumatized teeth is the accumulation of haemoglobin or other forms of haematin molecules. In the absence of bacterial invasion, it is unlikely that the protoporphyrin ring could be disintegrated to permit the release of iron. Histological examination of the teeth discolored by products of erythrocytes indicated that the blood pigments responsible for the discoloration are found within the dentinal tubules and not in the intertubular dentine. Erythrocytes do not contain self-digesting autolytic enzymes but require a normal inflammatory response to allow the breakdown of the haemoglobin, however, the pulp chamber is surrounded by dentine and cementum, which isolates it from any inflammatory or healing response of adjacent tissues [60].

Previous studies have shown that contamination with blood increases discoloration associated with MTA and CSCs. [2,3,22,23,25,26,27,42,61]. This is in agreement with our findings of this review regarding Biodentine. Histochemical tests have shown a color gradient in teeth stained with erythrocytes, with the stain being greatest in the dentin closest to the pulp chamber and decreasing in intensity outward [2,60]. The Lenherr et al. [3] study showed that Portland cement–based materials, such as the white and gray ProRoot MTA and Portland cement, were associated with greater color changes after blood contamination. The Felman et al. [2] study revealed that blood contamination of white ProRoot MTA increases its degree of discoloration. Shokouhinejad et al. [23] and Chen et al. [25] studies demonstrated that blood contamination caused similar discoloration for all materials tested. Some previous studies also found that irrespective of the type of material, blood contamination may increase discoloration over time [21,23,25,26,27,42]. The exact mechanism for intensified discoloration of CSCs in the presence of blood has yet to be fully understood [2,11]. One of the hypotheses to explain the mechanism of more severe tooth discoloration in the presence of blood may be related to the oxidation and incorporation of the remaining iron content within the wMTA powder into the calcium aluminoferrite phase of the set wMTA cement [2]. Alternative materials to MTA have been developed and the release of heavy metal ions similar to MTA has been reported, although their effect on tooth discoloration is unknown, but conceivably minimal, because of the short-term release [2]. Another possible mechanism may be the interaction between the erythrocytes and the unset wMTA. The slow hydrating process of wMTA may permit the penetration of erythrocytes from the adjacent pulpal tissue into teeth structure and calcium silicate-based material. Furthermore, hemolysis of penetrated red blood cells may result in both material and subsequent tooth tissues’ discoloration [2,7,11,22]. Moreover, from previously mentioned studies it can be concluded that the presence of blood during the setting of CSC can exacerbate both MTA and Biodentine color variation over time. On the other hand, blood can induce discoloration due to the penetration into the pores of the cements [3,7,21,26]. It has been shown that voids and pores may entrap blood components and cause discoloration of the material [3,62]. Longer setting time may be related to material remaining porous for a longer time, which results in increased blood absorption and subsequent hemolysis and greater discoloration [21,26]. Other studies demonstrate that Biodentine has lower discoloration potential than MTA or TotalFill in the presence of blood [8,26,27]. However, Adl et al. [42] at 3 months, Palma et al. [26] at one week and six months, and Keskin et al. [40] as well as Chen et al. [27] at all measuring time points, found Biodentine induced a human eye perceptible color change in the presence of blood. Moreover, Al-Hiyasat et al. [13] stated, that Biodentine was the most affected by blood contamination, in comparison with grey MTA, wMTA, TotalFill, and TheraCal. The authors suggest that this may be due to the fact that Total Fill and TheraCal are premixed materials and as such, can present better homogeneity, which may be in contrast to Biodentine, as it has to be mixed in an amalgamator before use. Due to this reason, the mixture may entrap blood molecules more easily, which can penetrate within the particles of the material, whereas this may be less likely in the premixed materials. However, in vitro studies have some limitations. Erroneous results may be made by the accidental mixing of the material with blood that could implicate the chromatic change of the material and hence the tooth [25]. In addition, another limitation of in vitro studies is the absence of positive pulp pressure, which might limit the blood flow towards the periphery [26]. In some studies, the control group with blood has shown the highest discoloration values, which could be explained by the material blocking the blood components’ influx [22].

Irrigation solutions used in endodontics have influence on the color of MTA and CSCs. The study by Camilleri et al. [16] evaluated the effect of routine solutions used in endodontics on Portland cement, wMTA, and bismuth oxide. It has been shown that the immersion of wMTA and bismuth oxide in sodium hypochlorite (NaOCl) resulted in the formation of a dark brown discoloration. Contact of bismuth-containing substances with NaOCl led to nearly black discoloration. The change in color could indicate a change from oxide to bismuth metal, which is black in color. The discoloration has been attributed to the destabilization of bismuth oxide in contact with a strong oxidizing agent [63]. Another hypothesis explaining the change in color would be the further oxidation of bismuth oxide that can cause destabilization of the oxide, with a reaction of carbon dioxide in the air leading to the formation of bismuth carbonate, which is light sensitive and forms black precipitate [16]. The X-ray diffractogram of bismuth carbonate has peaks in similar locations to bismuth oxide, making its detection difficult [63]. A similar study by Marciano et al. [63] examined bismuth oxide, calcium tungstate, and zirconium oxide placed in contact with NaOCl. Furthermore, bovine teeth previously immersed in water or NaOCl were filled with MTA Angelus, Portland cement, Portland cement with zirconium oxide, or calcium tungstate and Biodentine. Similar to the results of the study by Camilleri et al. [16], bismuth oxide in contact with sodium hypochlorite exhibited a change in color from light yellow to dark brown. The energy-dispersive maps showed migration of radiopacifier and silicon in dentine. This was verified where tooth discoloration was demonstrated when MTA Angelus was used in contact with dentine. The results of SEM mapping also indicated the migration of the NaOCl and evaluated cements within dentine. The radiopacifier particles, bismuth, zirconium, and tungsten were also identified in dentine. The presence of bismuth in dentine and also the elemental components of sodium hypochlorite and their interaction are responsible for causing discoloration. Zirconium oxide and calcium tungstate, although exhibiting migration in dental tissues, have not been implicated in color alteration. The prototype cements and Biodentine did not show any related dental discoloration. Another Camilleri et al. study [29] aimed to characterize Neo MTA Plus, MTA plus, and Biodentine and assess their color stability in the presence of NaOCl solution. Studies agree that the bismuth oxide reaction with NaOCl is responsible for dental tissue color alterations [16,29,30,63]. Only MTA Plus contains bismuth oxide and exhibited discoloration, whereas Neo MTA Plus and Biodentine do not include bismuth oxide and do not exhibit discoloration [29]. On the contrary, the study of Keskin et al. [64] evaluated color stability of different calcium silicate-based materials in 24 h contact with different irrigation solutions using a spectrophotometer. All materials including Biodentine exhibited clinically perceptible discoloration when immersed in NaOCl and chlorhexidine gluconate (CHX). Biodentine exhibited more discoloration when immersed in CHX compared with NaOCl. The mechanism of material discoloration by CHX is not well-defined and may be related to surface microstructure. The study summarized that calcium silicate-based materials without bismuth oxide could be an alternative to MTA; however, in this study, all calcium silicate–based materials exhibited clinically perceptible color changes. Distilled water did not result in the discoloration of any materials tested [64].

EDTA removes the smear layer and consequently opens the dentinal tubules, increasing the contact of cement with dentin [9]. The severity of tooth discoloration depends on whether or not the smear layer is removed [65]. It has been reported that the smear layer can markedly reduce the permeability of dentine. In studies performed without the removal of the smear layer, tooth discoloration was less evident or took longer [3,65]. The discoloration severity increased by blood might be related to absence of the smear layer, which can reduce or increase dentin permeability [26]. From the articles included in this systematic review, all studies except two [14,22] used sodium hypochlorite as the main irrigant in concentrations varying from 0.5% to 6% [21,23,24,25,26,38] followed by irrigation with EDTA 17% and NaOCl for the final flush. However, two studies of Ramos et al. [22] and Nagas et al. [14] did not use irrigation. The Ramos et al. [22] study results indicated perceptible color change at 6 and 52 weeks for Biodentine, with positive and negative controls. However, Biodentine-induced discoloration was lower than negative control. Nagas et al. [14] study did not reveal perceptible color change (∆E ≥ 3.3) at any time interval.

Another factor, which may have an impact on discoloration potential, is the type of endodontic access cavity. In some studies, coronal access cavities were made [12,14,21,22,23,24,25,28,41], while in others retrograde [13,26,27,42] access cavities were prepared via apical access to the pulp chamber. Retrograde access cavity is not similar to clinical conditions and makes it difficult to remove residual pulp tissues from the pulp chamber and pulp horns [21]. However, this method provides a closed system that prevents the potential complication of coronal microleakage and also allows standardization of the thickness and volume of the tested materials [42]. When the occlusal access cavities were prepared, they were filled with composite resin after the application of CSCs. Although preparing an access cavity is similar to the clinical situation, filling the access cavities with composite resin may affect the results of studies [42]. The Madani et al. [21] study examined the effect of access cavity preparation on tooth discoloration; comparison of ΔE before and after the cavity preparation showed slight color change, but this difference was not statistically significant (*p* > 0.05).

Nevertheless, there are quite a few limitations regarding this systematic review. First of all, there is a high heterogeneity of methodologies of the selected studies, including sample sizes, sample preparation, irrigation protocols, methods and accuracy of evaluations, and follow-up time. Sample sizes varied from 7 (Ramos et al. [22]) to 30 (Adl et al. [42]) per group, which may be an important drawback evaluating strength of the gathered evidence. High variability, in particular, can be noticed regarding follow-up time, which varied, as in some articles it was measured up to 52 weeks (Ramos et al. [22]) and 2 years (Nagas et al. [14]), while in others up to 6 months (Valles et al. [27], Chen et al. [25], Palma et al. [26]), 4 or 3 months (Marques et al. [12], Adl et al. [42], Araghi et al. [41]), and only 1 month (Keskin et al. [40]). Furthermore, time points, at which measurements were taken, differ among the studies. Lack of standardization in these aspects prevents accurate interpretation and conclusions of the possible effect of Biodentine discoloration potential, as time appears to be an important factor for changes of teeth color and its degree to appear and be noticed.

Another limitation of this review is that only in vitro studies were included. To the authors’ knowledge, a few in vivo studies are available regarding this topic (Parinyaprom et al. [31], Abuelniel et al. [33], Linu et al. [34], Aly et al. [35], Haikal et al. [36], Uesrichai et al. [37]). However, they were not included in this review, as they did not meet inclusion criteria, mostly due to the fact that a reliable color determination method was not used. This, together with previously mentioned drawbacks of in vitro studies, highlights important criteria that should be met in future research regarding this topic—high quality in vivo studies with strict and homogeneous methodologies, longer follow-up periods, and reliable color determination methods are needed in order to draw clear conclusions about Biodentines’ discoloration potential and its relevance in clinical practice.

## 5. Conclusions

Overall, within the limitations of this systematic review, it can be concluded that direct contact with blood during dental procedures may enhance teeth discoloration when Biodentine is used. However, as results of in vitro studies are contradictory, the effect of Biodentine regarding color changes in the absence of blood is still unclear, as it is not clear what clinically relevant results could be expected in regard to the discoloration frequency and intensity induced by Biodentine. Therefore, more standardized in vivo studies with long periods of follow-up are needed.

## Figures and Tables

**Table 1:** Extracted from the Included Studies.

Nr	Lead Author	No. of all Tested Teeth, Teeth in Biodentine, Control Groups	Type of Teeth	Control	Tested Materials	Methods	Irrigation	Colour Measuring Method	Measuring Time	Human Eye Perceptible Colour Change
1	Madani et al. (2019)	68, 20 (10 saline, 10 blood), 8	Single rooted anteriors	4 teeth saline, 4 teeth blood, restored with composite	MTA-Angelus, CEM cement, Biodentine	Apical portion of the root sectioned, 5 mm of the root remained. Standart access cavities prepared, canals shaped by #1-6 Gates Glidden Drills. End of the root filled with Composite resin. Sponges with blood or normal saline embedded to CEJ. 3 mm of tested cement were placed in the access cavity on the sponges. After 24 h all the cavities sealed with composite resin by 2 mm. Specimens were kept in an incubator at 37 °C and 100% humidity.	5.25% NaOCl, saline	Spectrophotometer (Vita Easy shade, Germany)	After praparing the cavity (baseline), 1 day, 1, 6 months	∆E ≥ 3.3
2	Adl et al. (2019)	70, 30 (15 saline, 15 blood), 10	Single rooted maxillary anteriors and mandibular premolar	5 teeth saline, 5 teeth blood	MTA-Angelus, Biodentine	Apical portion of the root sectioned, 10 mm from buccal CEJ remained. Canals prepared from apical aspect to pulp chamber roof (closed system). Canals enlarged with #2-6 Gates Glidden drills, last instrument was ParaPost drill size 7 (1,75 mm diameter). Tested materials packed to buccal CEJ. Cotton pallets placed from apical aspect with normal saline or blood. Apical openings sealed with sticky wax. Every specimen was placed into a single vial-containing 1 mL of saline as the	2.5% NaOCl, 17% EDTA, saline	Spectrophotometer (Spectroshade MHT S.p.A., Verona, Italy)	Before materials placement, 1 week, 1, 3 months	ΔE > 3.7
3	Chen et al. (2020)	100, 20 (10 saline, 10 blood), 20	Single canal anteriors	10 positive control with blood clot up to CEJ, 10 negative control with only saline	EndoSequence RRM putty, EndoSequence RRM fast set putty, Biodentine, ProRoot WMTA	Apical portion of the root sectioned, 10 mm apical from buccal CEJ remained. Coronal access prepared, root canals enlarged with Gates Glidden drills #4. Cotton pallet was placed down to the CEJ, the root ends were sealed with composite resin. Canals filled with blood approximately 4 mm below buccal CEJ, the blood was allowed to clot for 15 min. 2 mm collagen plug placed, then 3 mm of tested materials packed. In the groups with no blood, collagen plug 3 mm below CEJ placed, tested materials placed over it. Access cavities sealed with temporary restorative material. Specimens were stored at 37 °C and 100% humidity.	Passive ultrasonic irrigation with 4% NaOCl, 17% EDTA, distilled water	Spectrophotometer (Ocean Optics, Dunedin, FL), A dental microscope (OPMI ProErgo, Carl Zeiss), high-definitioncamera (Nikon D700, Nikkor 105 mm f/2.8GIF-ED; Nikon, Tokyo, Japan)	Baseline (before appliacation of cement) Day 0, 1, 2, 6 months	∆E > 3.3
4	Shokouhinejad et al. (2016)	104, 24 (12 saline, 12 blood), 8	Single rooted anteriors	4 saline, 4 blood	Biodentine, OrthoMTA, EndoSequence Root Repair Material, ProRoot MTA	Apical portion of the root sectioned, 5 mm apical of roots remained. Coronal access cavities prepared. Root canals were cleaned and shaped using #1 to 6 Gates Glidden drills. Customized cylindrical piece of plastic white foam was inserted into the root canal through the apical opening up to CEJ of the labial surface. Apical part sealed with composite resin. The inserted foam was saturated with fresh human blood or normal saline. 3 mm of tested materials placed inside access cavities, temporary restored with Coltosol, incubated at 37 °C in fully saturated humidity for 24 h, later restored with composite resin.	5.25% NaOCl, 17% EDTA	Konika CS2000 spectroradiometer (Minolta, Osaka, Japan)	Before materials placement, 24 h, 1, 6 months	NM
5	Palma et al. (2020)	54, 12 (6 saline, 6 blood), 6	Premolars	3 saline, 3 blood	ProRoot MTA, Biodentine, TotalFill BC putty, pulp capping material	Apical portion of the root sectioned, 2 mm apical from CEJ remained. Access cavity was prepared through root-end preparation. Cavities centered on the pulp chamber with 4 mm depth and 2 mm diameter were obtained, ensuring a peripheral minumum of 1 mm enamel and dentin. Cavities were filled with a sterile cotton pellet moistened with saline solution or blood and tested materials inserted into cavities directly over the liquid solutions. All cavities filled with composite resin. Specimens were kept in thedark environment, in an incubator at 37 °C and 100% humidity.	2.5% NaOCl, 17% EDTA, saline	Canon EOS 5DsR camera, Color assessment was performed ImageJ (National Institutes of Health, NIH) software	Baseline (after cavity preparation), immediately after biomaterial placement and restoration, after 72 h, 7 days, 6 months	∆E ≥ 3.3
6	Al-Hiyasat et al. (2021)	144, 24, 24	Maxillary premolars	Nagative with saline and 2 mm composite, positive control with blood and 2 mm composite	GMTA Angelus, ProRoot WMTA, Biodentine, TheraCal, TotalFill	Apical portion of the root sectioned, 1 mm apical from CEJ. Pulp chambers were prepared to a standard size 5 mm depth and leaving 3 mm of buccal thickness. Then 3 mm of tested materials were covered with saline or blood. 2 mm of cavites filled with composite resin. Each tooth was then stored separately in a labelled plastic cuvette in an incubator at 37 °C in 100% humidity in distilled water.	2.5% NaOCl, normal saline	Spectrophotometer (VITA Easyshade compact; VITA Zahnfabrik, Bad Säckingen, Germany)	Prior material placement, 24 h, 1 week, 1, 3, 6 months	∆E ≥ 3.7
7	Marques Jr. et al. (2021)	40, 10, 10	Mandibular premolars	With temporary filling	WMTA Angelus, MTA Repair HP (Angelus), Biodentine	Apical portion of the root sectioned, 7 mm apical from CEJ. A conventional access cavity was prepared and the root canals were shaped by #1-6 Gates Glidden Drills. Root apex was sealed with 2 mm composite resin. Root canals were filled with blood until the CEJ, after 15 min tested materials were placed with a thickness of 3 mm. The access cavity sealed with flowable composite resin. Specimens were stored immersed into distilled water at 37 °C.	2.5% NaOCl, 17% EDTA, 2.5% NaOCl, distilled water	Sprectrophotometer (VITA Easyshade^®^ Advance 4.0; Vita Zahnfabrik, Bad Sackingen, Germany)	Before cavity preparation, after procedure, 1, 2, 3, 4 months	Perceptibility threshold ΔEab = 1.2, acceptability threshold 2.7
8	Keskin et al. (2016)	60, 20	Incisors	Baseline	ProRoot wMTA, Biodentine, BioAggregate	Root tips were resected from 3 mm apical level. Root canals were prepared with Gates Glidden drills #2-6, Rely-X Drill size 3 (1.9 mm diameter) until pulp chamber level. Materials compacted to pulp chamber and coronal portion of root canal to a thickness of 4 mm. All materials were compacted with EndoActivator. Cotton pellets were placed into the root canals via apical aspect and was saturated with blood. Apical accesses were sealed with light-cured glass ionomer cement. Specimens were stored in an incubator at 37 °C and 100% humidity.	5.25% NaOCl, 17% EDTA	Spectrophotometer (VITA Easyshade^®^ compact; VITA Zahnfabrik, Bad Sackingen, Germany), digital camera (Sony Cybershot DSC-W220, Tokyo, Japan)	Before application of materials as baseline, 24 h after, at 30 days	∆E ≥ 3.3
9	Marconyak Jr. et al. (2016)	90, 15, 15	Mandibular third molars	Negative control no treatment, ProRoot MTA positive	ProRoot MTA, ProRoot wMTA, EndoSequence Root Repair Material, MTA Angelus, Biodentine	Standart coronal access cavities, buccal enamel dentin chickness 3 mm, materials placed 3 mm thickness above orifice level and allowed to set. A 3 mm thickness of glass ionomer was placed over each material and allowed to set. Access cavities filled with composite, shade matched to the coronal tooth structure. Specimens were stored separately in phosphate buffered saline solution at 37 °C and 100% humidity.	6% NaOCl	Spectrophotometer (VITA Easy Shade; VITA Zahnfabrik, Germany), digital camera (Nikon D80; Nikon, Tokyo, Japan)	Baseline, after access preparation, after material placement, 1, 7, 30, 60 days	∆E ≥ 3.3
10	Kohli et al. (2015)	80, 10, 10	Single canal maxillary anteriors	No filling (negative control)	EndoSequence RRM putty, EndoSequence RRM fast set paste, Biodentine, white MTA, grey MTA, AHPlus sealer, TAP	Apical portion of the root sectioned, 10 mm apical from buccal CEJ remained. Coronal access cavities prepared, root canals were instrumented to a final size 60/04. EndoSequence Ni-Ti rotary files. Moist cotton pellet was placed from access cavity, plugged to the CEJ, entire coronal pulp chamber filled with temporary restorative material. Canals filled with tested material from the apical access, test material did not encroach on the pulp chamber. Tested materials was placed 6 mm length from CEJ to apical extent. Remaining canal sealed with Cavit. Specimens were stored in an incubator in 100% humidity at 37 °C.	Passive ultrasonic irrigation with 3% NaOCl, 17% EDTA, distilled water	Spectrophotometer (Ocean Optics, Dunedin, FL). Photographs were taken using a dental operating microscope (Carl Zeiss OPMI ProErgo) with an internal high definition camera at 7.5 magnification Spectrophotometer (SpectroShade, Handy Dental Type 713000; MHT, Arbizzano di Negar, Verona, Italy)	Day 0 (after tooth prepation, before placement of materials), 7, 1, 2, 6 months	∆E ≥ 3.7
11	Valles et al. (2015)	35, 16, 3	Single rooted. Each group: 8 maxillary, 8 mandibular teeth, equal numbers of central, lateral incisors, canines	Composite alone	ProRoot WMTA, Biodentine	Apical portion of root sectioned, 1 mm apical from CEJ of root remained. Retrograde access cavity prepared 2 mm to incisal edge. Tested materials placed into cavities and not sealed. After 48 h sealed with composite. Specimens were kept at room temperature at 100% relative humidity and below a compact fluorescent lamp.	4.2% NaOCl, saline	Spectrophotometer (SpectroShade, Handy Dental Type 713000; MHT, Arbizzano di Negar, Verona, Italy)	After material placement, 1, 2 weeks, 1, 3, 6 months	NM
12	Araghi et al. (2019)	64, 20, 4	Single rooted. Each group: 8 maxillary, 8 mandibular teeth, equal numbers of central, lateral incisors, canines	No cement after root canal treatment	MTA-Angelus, Biodentine, CEM cement	Standart access cavities prepared. Root canals enlarged with Gates Glidden drills #1-3, hand K-files, Mtwo (VDW, Germany) rotary files, then filled with gutta-percha and AH26 sealer using lateral compaction technique, gutta-percha was cut at the level of orifice. 3 mm of tested cement applied, teeth restored with composite matching teeth color. Specimens were immersed in saline and stored at room temperature under natural lighting. Saline solution was refreshed every 3 days.	Saline, 0.5% NaOCl	Spectrophotometer (Vita EasyShade^®^ compact, VITA Zahnfabrik, Germany)	Baseline (before application of cement), 1 week, 1, 2, 3 months	NM
13	Nagas et al. (2021)	64, 20, 4	Third molars	Negative control no cement, intact teeth	ProRoot MTA, MTA Angelus, NeoMTA, EndoSequence Putty, Biodentine	Coronal endodontic access cavities were made, coronal pulps were removed. Tested material placed 3 mm-thick on the pulp chamber floor. Thin layer of glass ionomer was placed over tested materials. Remaining access cavity was restored with acid-etch composite matching color with coronal tooth stucture. Specimens were stored separately in phosphate buffered saline solution at 37 °C in 100% humidity.	Non	Spectrophotometer (Spectroshade MHT S.P.A., Verona, Italy) and digital images with Canon EOS 650D	Baseline before endodontic access, immediately after placement,	∆E ≥ 3.3
14	Ramos et al. (2016)	28, 7, 14	Premolars	7 negative (dry cotton pallets), 7 positive (fresh blood cotton pallets)	ProRoot MTA, Biodentine	Apical portion of the root sectioned, 2 mm apical from CEJ remained. Pulp tissues extirpated via the cervical cut with Hedstrom files. Access cavities prepared, tested material plugs 2 mm diameter and 5 mm height placed. For control groups cotton pellets, dry or moistened with blood, placed into the cavities. All cavities sealed with glass ionomer cement. specimens were stored in the dark in a 100% humidity at 37 °C with normal atmospheric gas levels.	Non	Colorimeter (PR-650 SpectraScan Colorimeter; PHOTO RESEARCH Inc, Chatsworth, CA, USA)	Baseline (after praparation of the cavities, before application of cement), after cavites sealing, 6, 52 weeks	∆E ≥ 2.3

**Table 2:** The Changes in Color (ΔE) Measured for Materials Tested at Different Time Intervals.

Nr	Lead Author	Human Eye Perceptible Color Change	Given Parameter	Group	Before Material Placement, Baseline	After Material Placement	72 Hours	1 Week	2 Weeks	1 Month	6 Weeks	2 Months	3 Months	4 Months	6 Months	52 Weeks	24 Months
1	Adl et al. (2019)	∆E > 3.7	ΔE	Biodentine	V			2.28 ± 1.62		2.38 ± 1.84			3.24 ± 2.23				
				Control saline	V			1.92 ± 1.59		3.23 ± 1.76			2.90 ± 1.70				
				Biodentine	V			3.20 ± 2.11		3.06 ± 2.38			**4.20 ± 2.89**				
				Control blood	V			**5.50 ± 4.21**		**6.00 ± 3.97**			**8.32 ± 5.78**				
2	Chen et al. (2020)	∆E > 3.3	∆E	Biodentine no blood	V			1.88 ± 1.06		1.99 ± 0.90		2.09 ± 0.95			2.29 ± 0.85		
				Control saline	V			1.29 ± 0.59		1.5 ± 0.42		1.7 ± 0.86			1.71 ± 0.34		
				Biodentine	V			**6.56 ± 2.87**		**6.46 ± 2.72**		**6.15 ± 1.80**			**6.82 ± 2.37**		
				Control blood	V			**14.3 ± 8.52**		**12.63 ± 10.5**		**11.09 ± 9.25**			**10.25 ± 9.89**		
3	Palma et al. (2020)	∆E ≥ 3.3	∆E	Biodentine	V	1.5 ± 0.6	1.4 ± 0.0	**3.4 ± 0.9**							**3.4 ± 0.9**		
				Control saline	V	**3.8 ± 2.1**	2.0 ± 0.0	2.2 ± 0.9							1.6 ± 0.6		
				Biodentine	V	1.6 ± 1.4	2.1 ± 1.0	**3.8 ± 1.2**							**4.6 ± 1.6**		
				Control blood	V	**4.4 ± 1.4**	**7.7 ± 4.0**	**6.7 ± 2.8**							**8.0 ± 1.7**		
4	Al-Hiyasat et al. (2021)	∆E ≥ 3.7	∆E	Biodentine	V			2.284 ± 1.171		2.290 ± 1.05			3.014 ± 1.403		3.290 ± 1.446		
				Control saline	V			1.669 ± 0.74		1.687 ± 0.685			2.339 ± 0.863		2.915 ± 0.606		
				Biodentine	V			**4.070 ± 1.19**		**4.488 ± 1.38**			**6.013 ± 1.896**		**6.681 ± 2.086**		
				Control blood	V			**8.041 ± 3.10**		**10.558 ± 2.7**			**11.965 ± 3.607**		**12.834 ± 3.935**		
5	Marques Jr. et al.	ΔEab = 1.2	∆E	Biodentine	V	**3.43 ± 192**				**10.27 ± 4.14**		**11.14 ± 4.2**	**11.98 ± 4.53**	**12.39 ± 4.39**			
				Control	V	**5.03 ± 3.42**				**3.83 ± 1.43**		**3.32 ± 1.92**	**3.85 ± 1.7**	**3.9 ± 1.82**			
6	Valles et al. (2015)	NM	∆E	Biodentine		V		2.78 ± 1.13	**3.76 ± 1.48**	**4.08 ± 1.75**			**4.19 ± 1.39**		**5.28 ± 2.12**		
				Control		V		2.88 ± 0.45	3.61 ± 0.89	**4.49 ± 0.69**			3.20 ± 0.71		**6.09 ± 1.15**		
7	Araghi et al.	NM	∆E	Biodentine	V	**8.03 ± 2.82**		**9.85 ± 3.35**		**10.12 ± 3.98**		**12.97 ± 4.95**	**13.01 ± 5.35**				
				Control	V	**10.51 ± 2.98**		**11.86 ± 0.73**		**17.01 ± 2.12**		**17.77 ± 1.85**	**17.81 ± 2.21**				
8	Nagas et al. (2021)	∆E ≥ 3.3	∆E S	Biodentine	V	1.80 ± 0.24							1.83 ± 0.46		1.89 ± 0.53	2.04 ± 0.47	2.28 ± 0.32
				Negative	V	1.34 ± 0.24							1.35 ± 0.46		1.58 ± 0.32	1.61 ± 0.47	1.68 ± 0.53
			∆E DC-	Biodentine	V	5.45 ± 0.93							5.66 ± 0.91		7.07 ± 1.06	7.39 ± 0.96	7.40 ± 0.88
				Negative	V	5.54 ± 0.91							6.51 ± 0.94		7.16 ± 1.06	7.17 ± 0.96	7.26 ± 0.88
			∆E DC+	Biodentine	V	6.35 ± 1.11							6.58 ± 1.15		6.93 ± 1.26	7.08 ± 0.80	7.23 ± 0.89
				Negative control	V	6 ± 1.11							6.05 ± 1.15		7.28 ± 1.26	8.23 ± 0.8	8.35 ± 0.89
9	Ramos et al. (2016)	∆E ≥ 2.3	∆E	Biodentine	V	1.25 ± 0.80					**3.72 ± 0.65**					**10.84 ± 1.95**	
				Positive	V	**3.41 ± 0.78**					**6.80 ± 1.81**					**14.53 ± 2.11**	
				Negative	V	1.14 ± 0.37					**3.92 ± 0.62**					**11.28 ± 2.11**	

V means baseline of ΔE calculations, ±Standard deviation, (S) Spectrophotometer, (DC-) Digital Camera without a cross-polarizing filter, (DC+) Digital Camera with a cross-polarizing filter.

## Data Availability

Not applicable.
